# Is thumb carpometacarpal instability assessed in young patients with thumb base symptoms? A multicenter study

**DOI:** 10.1016/j.jpra.2026.08.008

**Published:** 2026-08-22

**Authors:** Niek J. Nieuwdorp, Maximilian Mayrhofer-Schmid, Reinhard M. Knerr, Caroline A. Hundepool, Abhiram R. Bhashyam, Neal C. Chen, J. Michiel Zuidam, Kyle R. Eberlin

**Affiliations:** a Hand and Arm Center, Department of Orthopaedic Surgery, Massachusetts General Hospital, Harvard Medical School, Boston, MA, USA; b Division of Plastic and Reconstructive Surgery, Department of General Surgery, Massachusetts General Hospital, Harvard Medical School, Boston, MA, USA; c Department of Plastic, Reconstructive and Hand Surgery, Erasmus MC, Rotterdam, the Netherlands; d Department of Hand, Replantation-, and Microsurgery, BG Klinikum Unfallkrankenhaus Berlin and Chair of Hand, Replantation-, and Microsurgery at the Charité Universitätsmedizin Berlin, Berlin, Germany; e Hand and Wrist Center, Xpert Clinics, the Netherlands

**Keywords:** Thumb carpometacarpal joint, Carpometacarpal instability, Diagnostic variability, Diagnostic workup, Thumb base pain

## Abstract

Thumb base complaints in younger patients are diagnostically challenging, and symptoms are commonly attributed to either carpometacarpal (CMC) osteoarthritis or ligamentous instability. These conditions require fundamentally different treatment strategies, making accurate diagnosis essential. The reported frequency of CMC instability varies considerably across clinical practices, possibly reflecting differences in diagnostic awareness rather than true differences in prevalence. This study therefore aimed to describe the diagnostic workup of younger patients with nontraumatic thumb base complaints, with a focus on the use and findings of instability testing. In this retrospective multicenter study of 85 patients younger than 45 years, instability testing was performed in only 39%, yet was positive in 75% when assessed. Patient characteristics were similar between tested and untested patients, suggesting that testing reflected variation in diagnostic practice rather than clinical suspicion. Positive instability tests were also observed in patients labeled as osteoarthritis, often without radiographic signs of degeneration. These findings suggest that CMC instability may be underrecognized in younger patients, and that variation in diagnostic practice likely contributes to its inconsistent recognition. Failure to recognize instability may lead to osteoarthritis-directed surgery in patients who could have been treated with joint-preserving approaches. Systematic assessment of CMC instability is therefore essential to ensure accurate diagnosis and guide optimal treatment in this population.

**Level of evidence:**

Level IV

## Introduction

Thumb base complaints in younger patients represent a diagnostically challenging presentation.^1^ In the absence of degenerative radiographic changes, identifying an underlying cause can be difficult. Symptoms are commonly attributed to early carpometacarpal (CMC) osteoarthritis or ligamentous instability.

CMC instability is increasingly recognized as a contributor to thumb base pain in young patients.^2^ Unlike osteoarthritis, which is characterized by cartilage degeneration, instability occurs without degenerative joint changes and results from insufficiency of stabilizing ligaments, leading to altered joint mechanics and synovitis.^1^ Distinguishing between these conditions is clinically relevant, as instability is managed by restoring joint stability, whereas osteoarthritis may require joint-sacrificing procedures such as trapeziectomy or prosthetic arthroplasty.^3^

The reported frequency of CMC instability varies considerably across clinical practices,^2^ possibly reflecting differences in diagnostic awareness and evaluation rather than true differences in prevalence. In other words, clinicians who routinely test for instability may identify it more frequently, while those who do not may attribute the same complaints to osteoarthritis. Whether inconsistent assessment contributes to underrecognition of instability in younger patients therefore remains an important question. The aim of this study was to describe the diagnostic workup of younger patients presenting with nontraumatic thumb base complaints, with a focus on the use and findings of instability testing.

## Methods

A retrospective multicenter study was conducted across six hospitals in the United States. Patients younger than 45 years presenting with nontraumatic thumb base complaints to hand surgeons between 2018 and 2023 were included, based on presenting complaint rather than final diagnosis. Patients with prior thumb base surgery or inflammatory arthritis were excluded. Electronic medical records were reviewed for documentation of instability testing, grind testing, imaging findings, diagnostic labels, and initial management. Instability was tested by stabilizing the trapezium and displacing the base of the first metacarpal in a dorsoradial direction.

## Results

A total of 85 patients were included (Table 1). CMC instability was recorded as a diagnosis in 13%. Patients diagnosed with CMC instability were generally younger and more often engaged in heavy physical work. CMC instability was more frequently diagnosed by plastic surgeons and more often assessed with MRI (Supplementary Digital Content 1).

**Table 1:** Demographic and clinical characteristics.

Variable	Characteristics (N=85)
**Patient demographics**	
Age (y), median [IQR]	41 [27-43]
Female sex, N (%)	63 (74)
**Clinical demographics**	
Symptom duration (m), median [IQR]	6 [3-21]
Second opinion, N (%)	5 (6)
Affected hand, N (%)	
Left	22 (26)
Right	35 (41)
Both	28 (33)
Dominant side affected, N (%)	61 (72)
Occupational intensity, N (%)	
Unemployed	8 (10)
Light physical labor (e.g., office work)	38 (46)
Moderate physical labor (e.g., shop work)	20 (24)
Heavy physical labor (e.g., construction)	17 (21)
Missing	2 (2)
Concurrent diagnosis, N (%)	26 (31)
Specialty of provider, N (%)	
Orthopedic surgery	76 (89)
Plastic surgery	9 (11)
**Diagnosis**	
Final clinical diagnosis, N (%)	
CMC osteoarthritis	34 (40)
Early CMC osteoarthritis	22 (26)
CMC pain / synovitis	18 (21)
CMC instability	11 (13)

*N* number of patients, *y* years*, IQR* interquartile range, *m* months, *CMC* carpometacarpal.

**Fig. 1:**
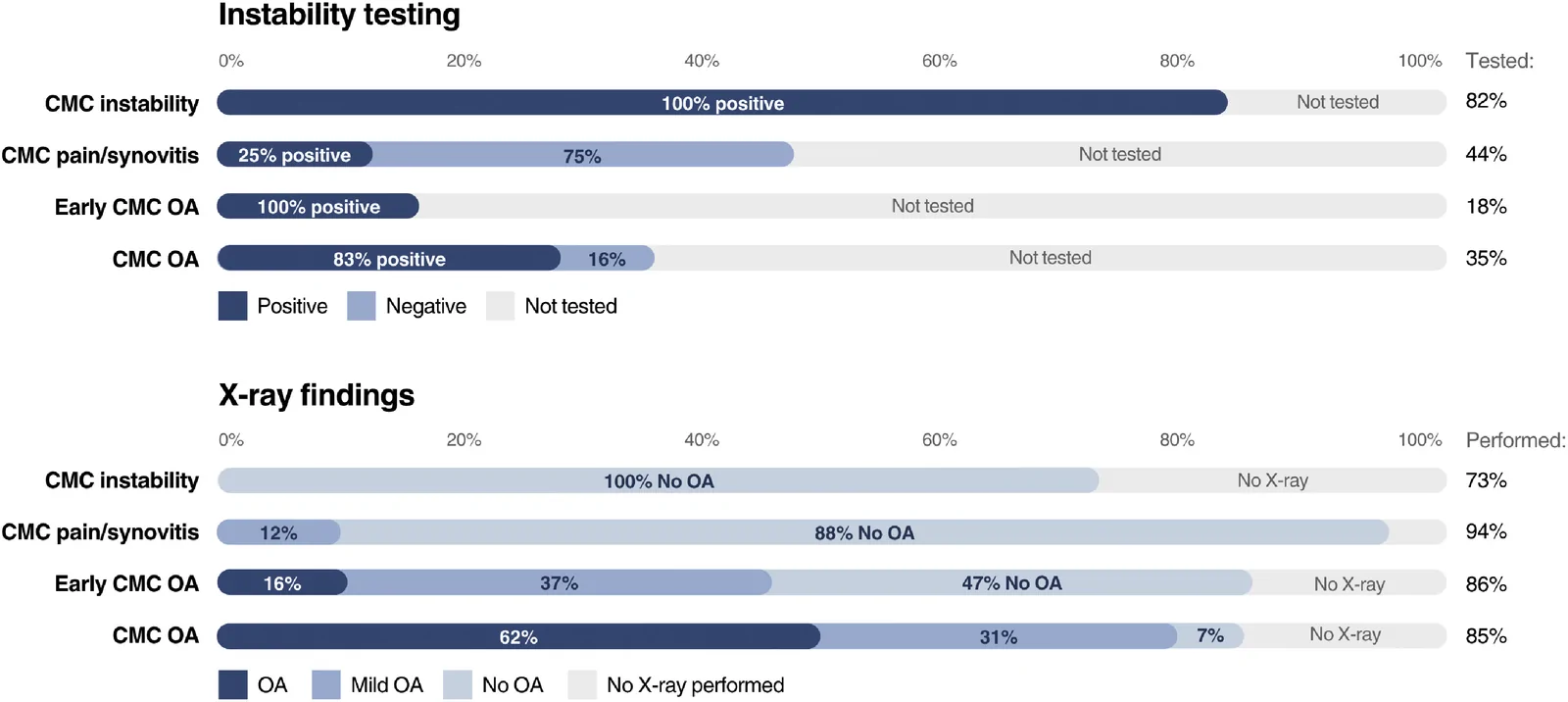
Clinical instability testing and radiographic findings stratified by final clinical diagnosis (left of each column). Instability testing was inconsistently performed but frequently positive across all groups, including those ultimately diagnosed as osteoarthritis (OA). Radiographic findings were assessed by the treating surgeon.

Clinical instability testing was performed in 39% of all patients and was positive in 75% when assessed. Patient characteristics were similar between tested and untested patients, suggesting testing was not guided by clinical suspicion but rather reflected variation in diagnostic practice (Supplementary Digital Content 2). Notably, positive instability tests were also observed in patients ultimately labeled as osteoarthritis (Fig. 1). The grind test was performed in 59% of patients.

Radiographs were obtained in 86% of all patients, showing absence of degenerative changes in 47%, mild changes in 25%, and osteoarthritis in 28%. Notably, a subset of patients diagnosed with early osteoarthritis showed no radiographic signs of degeneration (Fig. 1). Radiographic subluxation was described in 15% of patients and was present in patients across diagnostic groups. MRI was performed in 10%. Initial management after diagnosis included splinting (87%), activity modification or exercise therapy through an occupational or physical therapist (25%), and corticosteroid injection (17%).

## Discussion

In this multicenter cohort, CMC instability testing was performed in a minority of patients yet was frequently positive when assessed. This suggests that instability may be underrecognized in younger patients with thumb base pain.

This high positivity rate is consistent with a recent large cohort study in which systematic application of a standardized diagnostic algorithm identified CMC instability in 74% of young patients with nontraumatic thumb base complaints.^4^ The similar positivity rate observed in our cohort when instability was assessed (75%) suggests that the condition is genuinely common in young patients referred for thumb base complaints, and that its underrecognition is more likely attributable to inconsistent evaluation than to low true prevalence.

Variation in diagnostic evaluation is clinically consequential given that instability and osteoarthritis require fundamentally different treatment strategies. Instability is managed by restoring joint stability through exercise therapy or ligament reconstruction, whereas osteoarthritis management targets degenerative joint disease and may involve joint-sacrificing procedures such as trapeziectomy or prosthetic arthroplasty. Ligament reconstruction yields favorable outcomes.^3^ Importantly, it does not preclude osteoarthritis-directed procedures if needed, whereas the reverse is not true. Failure to recognize instability may therefore lead to joint-sacrificing surgery in patients who could have been treated with joint-preserving approaches. This is particularly relevant in younger patients, in whom trapeziectomy is associated with higher reoperation rates^5^ and worsening pain and functional decline over time. Long-term outcomes of prosthetic arthroplasty in this age group are not yet established. Because joint-preserving treatment does not preclude later joint-sacrificing surgery, it is the preferable first approach in young patients with thumb base pain.

The multicenter design of this study strengthens the generalizability of these findings. Reliance on documentation may have led to underreporting, however the high positivity rate supports the clinical relevance of our finding. This underscores the need for standardized diagnostic criteria to ensure consistent evaluation.

**Conclusion**

CMC instability is infrequently assessed in younger patients with thumb base pain despite frequent positive findings when evaluated. Differences in diagnostic workup likely contribute to inconsistent recognition. Given its distinct management from osteoarthritis, these findings underscore the importance of systematically assessing CMC instability to ensure accurate diagnosis and guide optimal treatment.

## Financial disclosure statement

Maximilian Mayrhofer-Schmid receives funding from the Deutsche Forschungsgemeinschaft (DFG, German Research Foundation) – Project number 566657538. Neal C. Chen is a consultant for Biedermann Motech. Abhiram R. Bhashyam is a consultant for Trimed, Depuy Synthes, Stryker, and RMD Medical. Kyle R. Eberlin is a consultant for AxoGen Inc., Checkpoint Surgical Inc., Integra Lifesciences Corporation, Tissium SA, Tulavi Therapeutics Inc., BioCircuit Technologies Inc., and Encoded Therapeutics Inc. The other authors have nothing to disclose.

## Ethical approval

Ethical approval for this study was obtained from the Institutional Review Board of Massachusetts General Hospital (IRB #2025P003167).

## Declaration of competing interest

None of the authors has a potential conflict of interest concerning this article's research, authorship, and/or publication.

## Appendix Supplementary materials

Image, application 1

Image, application 2

Image, application 3
