# Supplementary material for: Is thumb carpometacarpal instability assessed in young patients with thumb base symptoms? A multicenter study
Source: JPRAS Open. 2026 Aug 22;52:240–3. doi: 10.1016/j.jpra.2026.08.008 (PMC13571726; doi:10.1016/j.jpra.2026.08.008)
Supplement: Supplementary file 1 [file mmc2.pdf]

Supplementary Digital Content 1: Characteristics of patients presenting with non-traumatic thumb base complaints, stratified by diagnostic subgroup.

**Variable**	**CMC instability (N=11)**	**CMC synovitis/pain (N=18)**	**Early CMC osteoarthritis (N=22)**	**CMC osteoarthritis (N=34)**	**SMD**
Age (y), median [IQR]	36 [32-41]	39 [34-43]	41 [39-43]	42 [39-44]	0.73
Female sex, N (%)	7 (64)	14 (78)	15 (68)	27 (79)	0.21
Symptom duration (m), median [IQR]	5 [3-13]	3 [2-12]	6 [3-12]	8 [4-24]	0.26
Second opinion, N (%)	2 (18)	0 (0)	1 (5)	2 (6)	0.37
Affected hand, N (%)					0.36
Left	2 (18)	3 (16)	7 (32)	10 (29)	
Right	4 (36)	10 (56)	7 (32)	14 (41)	
Both	5 (46)	5 (28)	8 (36)	10 (29)	
Dominant side affected, N (%)	9 (82)	15 (83)	16 (73)	21 (62)	0.28
Occupational intensity, N (%)					0.60
Unemployed	1 (9)	0 (0)	0 (0)	7 (21)	
Light physical labor (e.g., office work)	4 (36)	11 (61)	12 (55)	11 (32)	
Moderate physical labor (e.g., shop work)	2 (18)	4 (22)	7 (32)	7 (21)	
Heavy physical labor (e.g., construction)	3 (27)	3 (17)	3 (14)	8 (24)	
Missing	1 (9)	0 (0)	0 (0)	1 (3)	
Concurrent diagnosis, N (%)	4 (36)	5 (28)	7 (32)	10 (29)	0.10
Specialty of provider, N (%)					
Orthopedic surgery	8 (73)	18 (100)	18 (82)	32 (94)	0.52
Plastic surgery	3 (27)	0 (0)	4 (18)	2 (6)	
Instability test performed, N (%)	9 (82)	8 (44)	4 (18)	12 (35)	0.65
Positive instability test, N (%)	9 (100)	2 (25)	3 (100)	10 (83)	1.27
Radiographs obtained, N (%)	8 (73)	17 (94)	19 (86)	29 (85)	0.31
Radiographic OA, N (%)					2.10
No OA	8 (100)	15 (88)	9 (47)	2 (7)	
Mild OA	0 (0)	2 (12)	7 (37)	9 (31)	
OA	0 (0)	0 (0)	3 (16)	18 (62)	
Radiographic subluxation, N (%)	5 (63)	0 (0)	5 (25)	1 (3)	1.00
MRI obtained, N (%)	3 (27)	1 (6)	2 (10)	2 (6)	0.33
Splint treatment, N (%)	8 (73)	17 (94)	19 (86)	30 (88)	0.32
Exercise therapy, N (%)	4 (36)	4 (22)	5 (23)	8 (24)	0.16
Injection performed, N (%)	2 (18)	4 (22)	4 (18)	4 (12)	0.14

N number of patients, *y* years*, IQR* interquartile range, *m* months, *OA* osteoarthritis.

SMD Standardized Mean Difference; around 0.2 indicates a small difference, around 0.5 a moderate difference, and around 0.8 a large difference.
Cohen J. *Statistical Power Analysis for the Behavioral Sciences.* Rev. ed. ed. Lawrence Erlbaum Associates; 1988
