# Supplementary material for: Is thumb carpometacarpal instability assessed in young patients with thumb base symptoms? A multicenter study
Source: JPRAS Open. 2026 Aug 22;52:240–3. doi: 10.1016/j.jpra.2026.08.008 (PMC13571726; doi:10.1016/j.jpra.2026.08.008)
Supplement: Supplementary file 2 [file mmc3.pdf]

Supplementary Digital Content 2: Characteristics of patients presenting with non-traumatic thumb base complaints, stratified by whether CMC instability testing was performed.

**Variable**	**Testing performed (N=33)**	**No testing performed (N=52)**	**SMD**
Age (y), median [IQR]	39 [36-42]	41 [38-44]	0.55
Female sex, N (%)	23 (70)	40 (77)	0.16
Symptom duration (m), median [IQR]	5 [2-24]	6 [3-18]	0.14
Second opinion, N (%)	2 (6)	3 (6)	0.01
Affected hand, N (%)			0.19
Left	7 (21)	15 (29)	
Right	15 (46)	20 (39)	
Both	11 (33)	17 (33)	
Dominant side affected, N (%)	25 (76)	36 (69)	0.15
Occupational intensity, N (%)			0.24
Unemployed	4 (12)	4 (8)	
Light physical labor (e.g., office work)	13 (39)	25 (48)	
Moderate physical labor (e.g., shop work)	9 (27)	11 (21)	
Heavy physical labor (e.g., construction)	6 (18)	11 (21)	
Missing	1 (3)	1 (2)	
Concurrent diagnosis, N (%)	6 (18)	20 (39)	0.46
Specialty of provider, N (%)			
Orthopedic surgery	28 (85)	48 (92)	0.24
Plastic surgery	5 (15)	4 (8)	
Radiographs obtained, N (%)	28 (85)	45 (87)	0.05

N number of patients, *y* years, *IQR* interquartile range, *m* months, *OA* osteoarthritis.

SMD Standardized Mean Difference; around 0.2 indicates a small difference, around 0.5 a moderate difference, and around 0.8 a large difference.

Cohen J. *Statistical Power Analysis for the Behavioral Sciences.* Rev. ed. ed. Lawrence Erlbaum Associates; 1988
