# Supplementary material for: Is thumb carpometacarpal instability assessed in young patients with thumb base symptoms? A multicenter study
Source: JPRAS Open. 2026 Aug 22;52:240–3. doi: 10.1016/j.jpra.2026.08.008 (PMC13571726; doi:10.1016/j.jpra.2026.08.008)
Supplement: Supplementary file 3 [file mmc1.docx]

**SUPPLEMENTARY DIGITAL CONTENT**

**Supplementary Digital Content 1.** Characteristics of patients presenting with non-traumatic thumb base complaints, stratified by diagnostic subgroup.

**Supplementary Digital Content 2.** Characteristics of patients presenting with non-traumatic thumb base complaints, stratified by whether CMC instability testing was performed.
